# Acute Stress in Lesser-Spotted Catshark (*Scyliorhinus canicula* Linnaeus, 1758) Promotes Amino Acid Catabolism and Osmoregulatory Imbalances

**DOI:** 10.3390/ani12091192

**Published:** 2022-05-06

**Authors:** Ignacio Ruiz-Jarabo, José A. Paullada-Salmerón, Ismael Jerez-Cepa, José Belquior Gonçalves Neto, Jason S. Bystriansky, Juan M. Mancera

**Affiliations:** 1 Departament of Biology, Faculty of Marine and Environmental Sciences, Instituto Universitario de Investigación Marina (INMAR), Campus de Excelencia Internacional del Mar (CEI-MAR), University of Cadiz, 11510 Puerto Real, Spain; joseantonio.paullada@uca.es (J.A.P.-S.); ismael.jerez@uca.es (I.J.-C.); belquior@alu.ufc.br (J.B.G.N.); juanmiguel.mancera@uca.es (J.M.M.); 2 Department of Physiology, Faculty of Biological Sciences, University Complutense, 28040 Madrid, Spain; 3 Instituto de Ciências do Mar, LABOMAR, Universidade Federal do Ceará, Fortaleza, CE 60165-081, Brazil; 4 Department of Biological Sciences, DePaul University, Chicago, IL 60614, USA; jbystria@depaul.edu

**Keywords:** air exposure, amiloride, amino acid consumption, elasmobranchs, NHE, osmoregulation, *Scyliorhinus canicula*, stress

## Abstract

**Simple Summary:**

In catsharks (*Scyliorhinus canicula*), air exposure induces amino acid catabolism altogether with osmoregulatory imbalances. This study describes a novel NHE isoform being expressed in gills that may be involved in ammonium excretion.

**Abstract:**

Acute-stress situations in vertebrates induce a series of physiological responses to cope with the event. While common secondary stress responses include increased catabolism and osmoregulatory imbalances, specific processes depend on the taxa. In this sense, these processes are still largely unknown in ancient vertebrates such as marine elasmobranchs. Thus, we challenged the lesser spotted catshark (*Scyliorhinus canicula*) to 18 min of air exposure, and monitored their recovery after 0, 5, and 24 h. This study describes amino acid turnover in the liver, white muscle, gills, and rectal gland, and plasma parameters related to energy metabolism and osmoregulatory imbalances. Catsharks rely on white muscle amino acid catabolism to face the energy demand imposed by the stressor, producing NH_4_^+^. While some plasma ions (K^+^, Cl^−^ and Ca^2+^) increased in concentration after 18 min of air exposure, returning to basal values after 5 h of recovery, Na^+^ increased after just 5 h of recovery, coinciding with a decrease in plasma NH_4_^+^. These changes were accompanied by increased activity of a branchial amiloride-sensitive ATPase. Therefore, we hypothesize that this enzyme may be a Na^+^/H^+^ exchanger (NHE) related to NH_4_^+^ excretion. The action of an omeprazole-sensitive ATPase, putatively associated to a H^+^/K^+^-ATPase (HKA), is also affected by these allostatic processes. Some complementary experiments were carried out to delve a little deeper into the possible branchial enzymes sensitive to amiloride, including in vivo and ex vivo approaches, and partial sequencing of a *nhe1* in the gills. This study describes the possible presence of an HKA enzyme in the rectal gland, as well as a NHE in the gills, highlighting the importance of understanding the relationship between acute stress and osmoregulation in elasmobranchs.

## 1. Introduction

Acute-stress responses in vertebrates include the release of catecholamines and corticosteroids into the blood [1,2]. These hormones increase heart and respiration rates, and gas exchange, and mobilize energy sources to fuel demanding tissues [3,4], preparing the body to fight, flight, and/or cope with the stressor. Minutes to hours after stressful events, changes in intermediary metabolism and osmo/ionic balance occur, many of which are related to the glucocorticoid (GC) effects of corticosteroids, while mineralocorticoids (MC) contribute to the recovery of homeostasis [5,6]. Despite the importance of unraveling the general physiological responses animals have to survive short-term traumatic events, knowledge gaps still exist, especially in more ancient species such as the elasmobranchs (including sharks and rays). In the lesser spotted catshark (*Scyliorhinus canicula* Linnaeus, 1758), an acute-stress challenge, such as air exposure, increases circulating levels of 1α-hydroxycorticosterone (1α-OHB) [7], the main corticosteroid in elasmobranchs [8]. Acute-stress challenges also induce osmoregulatory imbalances and blood acidification in several shark species [9,10,11], which may be related to the MC function of 1α-OHB [12,13]. It was postulated that 1α-OHB is involved in stress responses that affect carbohydrate metabolism [7], and GC actions mediated by corticosteroid-sensitive GC receptors have recently been described in *S. canicula* [14]. Although the metabolism of sharks following acute stress has been extensively studied [9,15,16,17], we consider it necessary to expand our understanding of the management processes of amino acids while recovering homeostasis following a stressful situation.

In this regard, amino acids are of paramount importance as oxidative substrates in white muscle of elasmobranchs. Their metabolism has some peculiarities, such as the ability to oxidize glutamine, which is an ancient strategy of vertebrates subsequently lost in tetrapods [18]. The unique intermediary metabolism of this group includes the absence of fatty acid oxidation in skeletal muscle, and a great reliance on ketone bodies along with amino acids as oxidative fuels [19]. Muscle produces ammonium (NH_4_^+^) along with alanine as the major nitrogen carriers to the liver, producing urea [20] and ketone bodies through hepatic amino acid turnover [21]. Excretion of nitrogenous compounds (urea and NH_4_^+^/NH_3_) occurs almost entirely at the gills, as described in spiny dogfish (*Squalus acanthias*), and has implications on acid-base balance and osmoregulation [22].

Marine elasmobranchs are slightly hypertonic with respect to the surrounding seawater due to the accumulation of urea in the body fluids, facilitating a net influx of water and diffusional gain of salts [23]. Osmoregulatory tissues, especially the rectal gland and the gills, expel the excess of ions [24,25], while the spiral valve and the rectum have a minor role in the maintenance of the osmo/ionic balance [26,27,28]. A basolateral Na^+^/K^+^-ATPase (NKA) is the major powering enzyme in the epithelium of these tissues, facilitating active ion transport [29]. 

Acute-stress responses in fish include osmotic imbalances, mostly due to increased permeability of the gills to improve gas exchange, allowing the passive diffusion of ions [17,25,30]. In this sense, increased blood osmolality occurs after capture in several shark species [9,31], including *S. canicula* [32], which is accompanied by increased levels of plasma Na^+^, Cl^−^, K^+^, and Ca^2+^ [10], followed by reduced urea levels in the blood [11]. An active Na^+^/urea-antiporter is present in gills of *S. acanthias*, which highlights the tight regulation of nitrogenous compounds and ion balance in sharks [33]. Moreover, it was described in *S. canicula* that branchial sodium uptake following an acute stress is associated with increased excretion of NH_4_^+^ and H^+^ ions [34]. Similarly, it was postulated in the teleost *Colossoma macropomum* that after an acute air-exposure challenge, the activity of a branchial amiloride-sensitive (ouabain/bafilomycin A1—insensitive) ATPase, that was described as a possible Na^+^/H^+^-exchanger (NHE), is related to blood NH_4_^+^ (as the end product of amino acid catabolism) and Na^+^ concentrations [35]. In mudskipper teleosts, branchial NHE may secrete NH_4_^+^ instead of H^+^ in exchange for Na^+^ [36], while in *S. acanthias* this was also suggested, along with the substitution of K^+^ by NH_4_^+^ on a branchial H^+^/K^+^-ATPase (HKA) transporter [37], previously described in elasmobranchs [38]. Several NHEs isoforms (NHE2 and NHE3) were described in gills of sharks and rays, along with other ion-transporters including the vacuolar-type H^+^-ATPase or VHA [39,40,41,42]. While their involvement in the acid-base and iono-regulation (especially NH_4_^+^ excretion) following acute-stress responses has been proposed, this needs to be fully confirmed in sharks.

The aim of this study is to gain further knowledge on the amino acid turnover, NH_4_^+^ excretion, and development of ion imbalances in shark plasma following an acute-stress challenge. We also sought to demonstrate tissue-specific ATPase activity relating protein metabolism to ion exchange, including the identification of novel enzymes in osmoregulatory tissues. The results will be discussed in terms of osmoregulatory management due to the enormous energy expenditure required to maintain a stable ionic balance. 

## 2. Materials and Methods

### 2.1. Ethics Statement

This study was performed in accordance with the Guidelines of the European Union (2010/63/UE) and the Spanish legislation (RD 1201/2005 and law 32/2007) for the use of laboratory animals. This study did not involve endangered or protected species. All experiments have been carried out under a special permit granted to the Spanish Institute of Oceanography, and approved by the Spanish General Secretariat of Fisheries (project DISCARDLIFE, Fundación Biodiversidad, Ministry for the Ecological Transition, Spain).

### 2.2. Air Exposure and Recovery Procedures

All maintenance and experimental procedures have been described in previous studies [7,32,43] and, in fact, the present study used some samples collected in those experiments. Therefore, we propose the comparison between the results derived from this study and those that have already been published. Lesser spotted catshark (*Scyliorhinus canicula*) adults and subadults of both sexes (*n* = 46, 380.6 ± 12.0 g body weight and 50.7 ± 0.5 cm total length, mean ± s.e.m) were obtained by bottom trawling as described previously [43] and maintained in the fish husbandry facilities of the Faculty of Marine and Environmental Sciences (University of Cadiz, Puerto Real, Cadiz, Spain) until the start of the experiment. Fish were randomly divided into six 400-L tanks (surface area of 0.72 m^2^, 0.56 m depth, and covered by overshadowing mesh). The tank system consisted of a flow-through supply of seawater (38 ppt), natural photoperiod (10:14, light:dark; November, latitude 36°31′34″ N), and temperature (19 °C) throughout the acclimation period (17 days). Fish were fed once per day at 20.00 UTC with fresh shrimp, prawns, sardines, and anchovies to satiety. Animals were fasted 36 h before sampling in order to avoid plasma imbalances related to feeding, as described previously in dogfish [26].

To evaluate the effects of air exposure, three tanks were selected as undisturbed controls, and catshark from the three other similarly sized tanks were used to expose fish to air. For this procedure, animals were captured by hand and placed in a dry tank for 18 min, then subsequently allowed to recover in water tanks. This process was previously described and induced acute stress responses in *S. canicula* [7,32]. Two or three animals from each tank (in triplicate, *n* = 7–8 per group) were sampled immediately after 18 min air exposure (without water recovery, 0 h), and 5 h and 24 h after air exposure. All sampling was conducted between 08.30–09.30 UTC. As anesthesia has been shown to affect stress-related blood variables in sharks [44], sampling was performed as follows. Catsharks were captured by hand while covering their eyes with a wet tissue (which calms the animal), blood was then immediately collected with a heparinized syringe from the caudal vessels (in less than one minute) and subsequently placed into heparinized tubes. Catsharks were then anesthetized in 0.1% *v/v* 2-phenoxyethanol (P1126, Sigma-Aldrich, St Louis, MA, USA). Weight and length of the animals was recorded. Catsharks were euthanized by severing the head with a sharp knife. All procedures lasted less than four minutes per tank. Plasma was then separated from cells by centrifugation of whole blood (3 min, 10,000× *g*, 4 °C) and snap frozen in liquid nitrogen. The second gill arch on the left side was excised, adherent blood was removed by blotting with absorbent paper and a small portion consisting of a few branchial filaments was cut using fine-point scissors. Each portion was placed in 100 μL of ice-cold sucrose-EDTA-imidazole (SEI) buffer (150 mM sucrose, 10 mM EDTA, 50 mM imidazole, pH 7.3) for the analysis of ATPases activity. The rectal gland was longitudinally divided into two aliquots with a sharp scalpel and one half was introduced into a tube containing 500 μL of ice-cold SEI buffer. A portion of the spiral valve (this was sectioned in half transversely, in the central part, and a sheet of about 2 mm thick was taken) was blotted with absorbent paper and placed in 500 μL of ice-cold SEI buffer. The remaining gill tissue, half of the rectal gland, spiral valve tissue, liver, and a portion of white muscle from the back (behind the first dorsal fin) were snap frozen in liquid nitrogen. All samples were stored at −80 °C until analyzed.

### 2.3. Plasma Parameters 

Sodium and potassium were measured by flame photometry (BWB-XP Performance Plus, BWB Technologies, Newbury. Berkshire, UK). Chloride was determined by potentiometric titration using the SAT-500 Analyzer (DKK-TOA, Tokyo, Japan). Plasma calcium, and magnesium levels were measured using commercial kits from Spinreact (Calcium ref. 1001061; Phosphate ref. 1001155; Magnesium ref. 1001280, Spinreact SA, Sant Esteve de Bas, Spain) adapted for 96-well microplates. The total plasma protein concentration was determined in diluted plasma samples using a bicinchoninic acid BCA Protein Assay Kit (Pierce, IL, USA, #23225) using BSA as a standard. Total α-amino acid levels were assessed colorimetrically using the described ninhydrin method [45] adapted to microplate assay. Plasma ammonium was measured following described methodologies [46], in which the NH_4_^+^ reacts with salicylate and hypochlorite to form a spectrophotometrically measurable product, a method validated for fish plasma samples and adapted to microplates [47]. The methodology employed to measure NH_4_^+^ has been chosen because the high levels of urea present in body fluids of elasmobranchs do not affect the measurement of ammonium [48,49,50]. However, we validated the methodology in a plasma-like medium which includes 385 mM urea (the composition of which is described in the section “Ex vivo gill incubations” below these lines) and decreasing concentrations of NH_4_^+^ from 8 to 0 μM. The results were compared with a similar dilution curve carried out in milli Q water. Plasma glucose, lactate, phosphate, and urea levels were measured using commercial kits from Spinreact (Glucose-HK ref. 1001200; Lactate ref. 1001330; Phosphate ref. 1001155; Urea ref. 1001323; Spinreact SA, Sant Esteve de Bas, Spain) adapted for 96-well microplates. All assays were performed using a Bio-Tek PowerWave 340 Microplate spectrophotometer (Bio-Tek Instruments, Winooski, VT, USA) using KCjunior Data Analysis Software for Microsoft Windows XP unless otherwise stated.

Osmolality was measured in 20 μL samples with a Vapor 5520 Osmometer (Wescor, USA). Plasma pH was measured immediately after centrifugation with a mini-electrode (HI1083B, Hanna Instruments, Woonsocket, RI, USA), and a 20 µL sample was collected for immediate analysis of total CO_2_ (TCO_2_) by means of an infra-red gas analyzer (IRGA, S151, Qubit systems, Kingston, ON, Canada) as described for other aquatic species [35,51]. Plasma pH and TCO2 analysis procedures were conducted at the acclimation temperature of catsharks. Calculations for plasma HCO_3_^−^ use described algorithms for dogfish [52,53] based on temperature and pH.

### 2.4. Tissue Metabolite Content

Frozen liver and muscle were finely minced on an ice-cooled Petri dish and divided into two aliquots to assess enzyme activities and metabolite levels. The frozen tissue used for the assay of metabolites was homogenized by ultrasonic disruption in 7.5 volumes ice-cold 0.6 N perchloric acid, neutralized using 1 M potassium bicarbonate, centrifuged (3 min at 10,000× *g* and 4 °C, Eppendorf 5415R), and the supernatant used to assay tissue metabolites. Total α-amino acid levels were assessed colorimetrically with the ninhydrin method of Moore [45] adapted to a microplate assay. 

### 2.5. Enzyme Activities 

Aliquots of liver, white muscle, gill, and rectal gland were homogenized by ultrasonic disruption in 10 volumes of ice-cold stop buffer (250 mM sucrose, 50 mM imidazol, pH 7.5, 1 mM 2-mercaptoethanol, 50 mM NaF, 4 mM EDTA, 0.5 mM PMSF, and a protease inhibitor cocktail—P2714, Sigma). The homogenate was centrifuged (10 min at 9000× *g*, 4 °C) and the supernatant used in enzyme assays for the analysis of amino acid-turnover metabolism enzymes such as GDH (glutamate dehydrogenase, EC 1.4.1.2), AST (aspartate aminotransferase, EC 2.6.1.1), ALT (alanine aminotransferase, EC 2.6.1.2), glycolytic-related enzymes like HK (hexokinase, EC 2.7.1.1), PK (pyruvate kinase, EC 2.7.1.40), and the phosphate shunt enzyme G6PDH (glucose-6-phosphate dehydrogenase, EC 1.1.1.49), as was described before for *S. canicula* [7] and other dogfish and elasmobranch species [54,55,56,57]. The reactions were started by addition of 15 μL homogenate at a pre-established protein concentration, omitting the substrate in control wells (final volume 275–295 μL). Data were expressed as U mg^−1^ prot.

Na^+^/K^+^-ATPase (NKA) activity in gill, rectal gland, and spiral valve homogenates were determined in microplates using a modification [58] of McCormick’s method [59] with 0.5 mM ouabain (O3125, Sigma-Aldrich) as a specific inhibitor of 100% NKA activity. Vacuolar-type H^+^-ATPase (VHA) activity was analyzed as described before [60], using 100 nM bafilomycin A1 (B1793, Sigma-Aldrich) as a specific inhibitor of the VHA. H^+^/K^+^-ATPase (HKA) activity was analyzed as described before [61], with 0.5 mM omeprazole (O104, Sigma-Aldrich) as a specific inhibitor of HKA activity. Amiloride-sensitive, ouabain and bafilomycin A1-insensitive, ATP-consuming enzymes, probably associated to a Na^+^/H^+^-exchanger (NHE), were analyzed using amiloride (A7410, Sigma-Aldrich) as an NHE (and Na^+^-channels) inhibitor [62,63] in the same biochemical conditions as for the other ATPase assays, as described before in a teleost species [35]. A concentration-response inhibition curve was performed covering a range from 1 µM to 500 µM amiloride. As the maximum inhibition occurs with 100 µM amiloride, this was the concentration routinely used in the assay. DMSO (<0.02% of the total incubation medium volume) was employed to dissolve omeprazole. Ouabain was combined with bafilomycin A1, omeprazole, or amiloride for the analysis of VHA, HKA, or amiloride-sensitive ATPase (NHE), respectively, in order to avoid activity overshadowing due to the high activity rates of the NKA. Data were expressed as µmol ADP mg^−1^ prot h^−1^. The reactions were allowed to proceed at 25 °C, similarly to what was described in *S. acanthias* [54]. Protein was assayed in triplicate as described for plasma samples. All enzyme activities were determined using a PowerWave^TM^ 340 microplate spectrophotometer (BioTek Instruments, Winooski, VT, USA) using KCjunior Data Analysis Software for Microsoft^®^ Windows XP. Reaction rates of enzymes were determined by changes in absorbance from the reduction of NAD(P)^+^ to NAD(P)H, measured at 340 nm.

### 2.6. Partial Molecular Cloning of Lesser Spotted Catshark nhe1 cDNA

The presence of NHE2 and NHE3 isoforms was described in gills of sharks [39,40,42,64,65], while NHE1 was also reported in branchial tissue of teleosts [66,67]. As NHE1, a phosphoprotein [68], was associated to ATP depletion and dependance [69,70,71], we explore the possible presence of this isoform in gills of *S. canicula*. Total RNA was extracted from a pool of lesser spotted catshark gills with TRIsure reagent (Bioline, London, UK) according to the manufacturer’s instructions. One microgram of isolated RNA was DNase I-treated (Ipswich, MA, New England Biolabs) and used to synthesize first-strand cDNA using iScript cDNA Synthesis Kit (Hercules, CA, Bio-Rad). Partial cDNA fragments were amplified by using the Q5^®^ High-Fidelity DNA Polymerase (New England Biolabs). The degenerate primers used to amplify the partial lesser spotted catshark *nhe1* were designed by means of ClustalW2 algorithm (http://www.ebi.ac.uk/Tools/msa/clustalw2/ accessed on 7 May 2019) from conserved regions among available vertebrate *nhe1* gene sequences (Table S1). The protocol used for *nhe1* PCR conditions were set as follows: 98 °C for 30 s followed by 35 cycles of denaturation at 98 °C for 10 s, 50–72 °C (optimized to the melting temperature of each primer pair used and according to the protocol’s instructions), extension at 72 °C for 30 s, and a final extension at 72 °C for 2 min. Amplified products were gel purified with QIAquick Gel Extraction (Qiagen, Chatsworth, CA, USA) and subcloned into pSpark II DNA cloning vector (Canvax Reagents, Córdoba, Spain) following the commercial protocols. Three positive clones were obtained by using the NEB 5-alpha Competent *E. coli* (High Efficiency, New England Biolabs) bacteria and sequenced in the Stab Vida Sequencing Service (Portugal) with vector-specific T7 and Sp6 primers. 

### 2.7. Nucleotide Sequence Analysis

The nucleotide and deduced amino acid sequences were carried out using the ExPASy translate tool (http://web.expasy.org/translate/ accessed on 14 June 2019) and the identity of partial lesser spotted catshark *nhe1* cDNA was confirmed by BLAST (http://blast.ncbi.nlm.nih.gov/Blast.cgi accessed on 17 October 2019). Transmembrane regions were determined using the Simple Modular Architecture Research Tool (SMART, http://smart.embl-heidelberg.de/ accessed on 20 May 2020). Phylogenetic analysis was accomplished using ClustalW (http://www.ebi.ac.uk/clustalw/ accessed on 20 May 2020) and the tree was conducted using the maximum likelihood method with MEGA version X [72,73].

### 2.8. In Vivo Amiloride Exposure

In order to assess the effect of in vivo inhibition of amiloride-sensitive enzymes (including NHEs) [62,63], catsharks were exposed to 100 µM amiloride (A7410, Sigma-Aldrich) as described for teleost fish, which inhibits 100% of NHE and Na^+^ channels [63,74]. *S. canicula* adults of both sexes (*n* = 53, 405.8 ± 10.9 g body weight and 49.4 ± 0.4 cm total length, mean ± s.e.m.) were obtained by bottom trawling as described before and maintained in onboard 350-L tanks for 24 h until the beginning of the experiment aiming at their physiological recovery, as described [32]. Once recovered, fish were exposed to air for 18 min mimicking the above described air-exposure experiment [7,32] and transferred individually to 8 L tanks (53 × 15 × 10 cm length, width, and height) with oxygenated clean water with or without 100 µM amiloride and maintained for 5 h. A control-undisturbed group (24 h + 5 h recovery in onboard tanks after capture by bottom-trawling; named as “sham” hereafter) and a group sampled immediately after 18 min air-exposure (0 h air, without recovery in water tanks) were included. Blood was collected (1 mL) after this time as described previously. All samplings were done at the same time of the day along 7 days, including 2–3 animals per group and day. No food was given to the catsharks during these procedures. Catsharks were released afterwards in the same geographical area as capture. Blood was also collected from 10 adult catsharks (5 females/5 males) immediately after bottom-trawling and before slaughtering by the fishermen (these stressed animals served as a control group and allow us to establish the maximum/minimum limits of variation of some plasmatic parameters) for further analysis of plasma parameters.

### 2.9. Ex Vivo Gill Incubations

To test the effects of those blood ions that were affected by in vivo air-exposure after 5 h recovery (plasma pH—H^+^, NH_4_^+^ and Na^+^, which are also the ions putatively exchanged by those ATP-consuming NHEs present in the gills), coinciding with changes in branchial amiloride-sensitive ATPase activity and NKA activity, gill filaments of *S. canicula* were ex vivo incubated. The incubation protocol was developed for liver explants for this species [14], and is based on previous protocols developed for teleost pituitary glands [75], and teleost fish gills [76,77]. An ad hoc incubation medium was developed for *S. canicula* after the analysis of plasma from control-undisturbed animals [14] maintained in similar conditions than those in the present study [7]. Control undisturbed catsharks (*n* = 8 mature females, 358.7 ± 13.3 g body weight and 50.8 ± 0.4 cm total length, mean ± s.e.m.) acclimated to our lab facilities (as described above in the “air exposure experiment”) were sacrificed, bled, and had gills removed with fine tweezers. Gill explants (4–5 filaments per sample) were maintained in 1 mL plasma-like incubation buffer for 60 min to accommodate fish needs, allowing the tissue to recover after the feasible stress induced by dissection. After this time, explants were transferred to 24-well microplates containing 1 mL fresh incubation medium (at a certain pH and/or NH_4_^+^ and Na^+^ concentrations, as described below) and incubated for 5 h (coinciding with the time at which *S. canicula* show highest amiloride-sensitive ATPase activity after in vivo air-exposure and recovery). All procedures were conducted at 25 °C. The incubation medium contained 210 mM Na^+^, 284 mM Cl^−^, 2.9 mM K^+^, 5.3 mM Ca^2+^, 2 mM Mg^2+^, 3.6 mM PO_4_^−^, 2 mM SO_4_^2−^, 3.4 mM HCO_3_^−^, 385 mM urea, and 2.3 mM glucose, and it was supplemented with 10 μL mL^−1^ vitamins (MEM 100× Vitamins, M6895, Sigma-Aldrich), 20 μL mL^−1^ essential amino acids (MEM 50×, M5550, Sigma-Aldrich), 10 μL mL^−1^ non-essential amino acids (MEM 100×, M7145, Sigma-Aldrich), 10 μL mL^−1^ antibiotics (penicilin 10,000 IU mL^−1^; streptomycin 10 mg mL^−1^, P0781, Sigma-Aldrich), and 20 μL mL^−1^ L-glutamine (200 mM, G7513, Sigma-Aldrich), adjusted to pH 7.8 with Trizma Base (T6791, Sigma-Aldrich) and osmolality of 925 mOsm kg^−1^ (with D-mannitol, M4125, Sigma-Aldrich).

The effect of plasma pH was tested at pH 7.8 (described in control-undisturbed catsharks), pH 7.0 (minimum plasma pH observed in this species, immediately after bottom trawling), and pH 8.0 (a higher pH than the control group). Tested NH_4_^+^ concentrations were 0.0 mM (control-undisturbed catsharks), 1.0 mM (after 18 min air-exposure in the first experiment of this study), and 5.0 mM (maximum concentration observed in *S. canicula*, immediately after bottom trawling). Tested Na^+^ concentrations were 210 mM (control-undisturbed catsharks), 220 mM (described in *S. canicula* exposed to air and after 5 h recovery), and 200 mM (as a lower Na^+^ concentration than the control group). Ammonium was added as ClNH_4_, and the final concentrations of Na^+^ and Cl^−^ in the incubation medium were adjusted with NaCl and choline chloride. The pH of the incubation medium was controlled and adjusted before the incubations. All experimental groups were tested in 8 animals, in duplicate each sample. Gill explants after 5 h ex vivo incubation were snap frozen in dry ice and maintained at −80 °C until the analysis of amiloride-sensitive ATPase and NKA activities.

### 2.10. Statistics

Normality and homogeneity of variances were analyzed using the Shapiro–Wilk´s test and the Levene´s test, respectively. The amiloride-inhibition of ouabain/bafilomycin A1-insensitive ATPase activity was evaluated using one-way ANOVA with amiloride concentration as the factor of variance. Differences between groups in the air-exposure and recovery experiment were tested using two-way ANOVA with group (control and air exposure) and time (0, 5, and 24 h) as the factors of variance. Differences due to amiloride in vivo exposure were tested using one-way ANOVA with group as the factor of variance. Differences due to ex vivo culture of gills were tested using one-way ANOVA with pH, or concentration of NH_4_^+^ or Na^+^ as the factors of variance. When necessary, data were logarithmically transformed to fulfill the requirements of ANOVA. Tukey´s post-hoc test was used to identify significantly different groups. A Linear Regression Model was performed to determine the correlation between parameters analyzed in the present study and those described in other published studies but analyzed on the same samples [7,32]. These correlations include the comparison between gill amiloride-sensitive ATPase activity and plasma Na^+^ or 1αOH-B (measured in a previous published study), and plasma Na^+^ and urea (measured in a previous published study), as described before in a teleost fish [35]. Statistical significance was accepted at *p* < 0.05. All the results are given as mean ± s.e.m.

## 3. Results

*p*-Values resulting from the two-way ANOVA of all assessed parameters from the air-exposure experiment are shown in Table S2. No mortality occurred during the experiments.

### 3.1. Amino Acid Turnover in Liver and Muscle after Air Exposure

**Figure 1:**
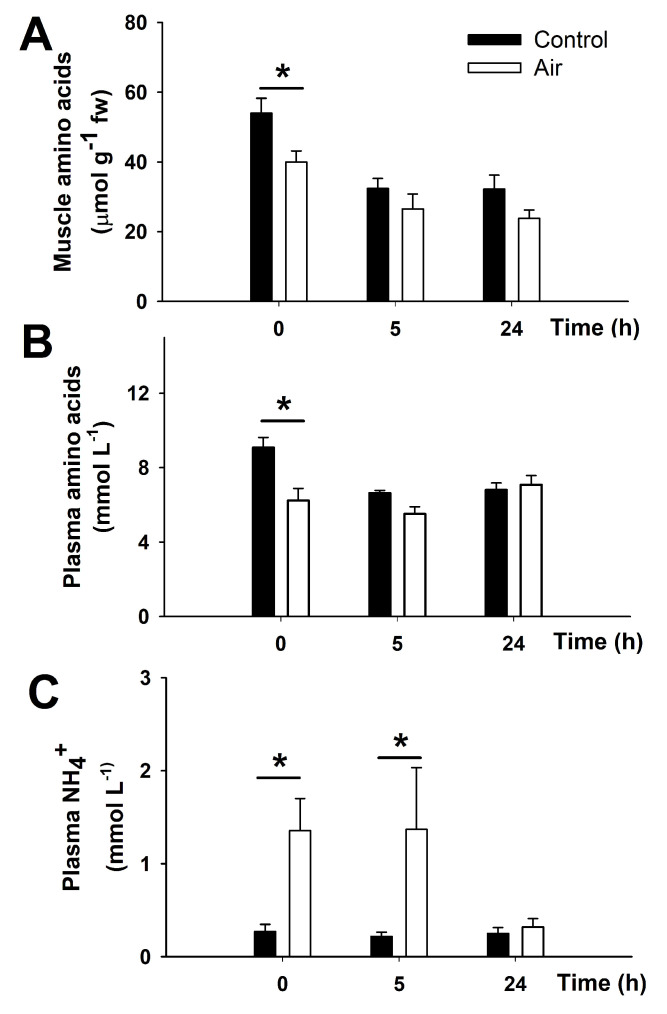
Amino acid consumption and NH_4_^+^ production in *S. canicula* after air exposure. Muscle free amino acids (in µmol g^−1^ wet weight; (**A**)), and plasma free amino acids (in mmol L^−1^; (**B**)) and NH_4_^+^ (in mmol L^−1^; (**C**)) in *S. canicula* after air exposure and recovery. Data are expressed as mean ± s.e.m. Asterisks (*) indicate significant differences between both groups at each time (*p* < 0.05, *n* = 7–8).

Free amino acid levels decreased in both muscle and plasma immediately following 18 min of air exposure (time 0 h). This coincided with an increase in plasma NH_4_^+^ at times 0 h and 5 h after recovery (Figure 1). Plasma measurement of NH_4_^+^ was not affected by the high urea concentrations observed in plasma of this species, as seen in the Supplementary material (Figure S1). There were no changes in plasma proteins or hepatic free amino acids during the time course in any of the groups tested (results not shown). Amino acid turnover-related enzymes such as glutamate dehydrogenase (GDH), and alanine and aspartate transaminases (AST and ALT) activities in white muscle and liver are shown in Table 1. Liver and muscle had significantly lower GDH activity in air exposed fish at time 5 h compared to control group. Muscle AST activity also decreased after 18 min of air exposure (time 0 h). No changes in muscle ALT or hepatic AST and ALT activities were found due to air exposure or time. Some differences in the control group were seen over the course of the experiment which may have been due to sampling time or time since last feeding. For this reason, all sampling points (0, 5, and 24 h) included the air-exposed group altogether with a control-undisturbed group.

### 3.2. Osmoregulatory Disturbances Following Air Exposure

**Figure 2:**
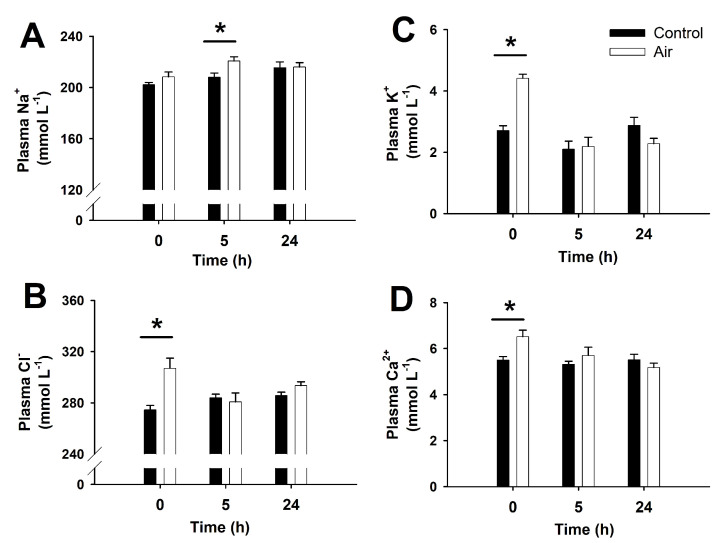
Plasma sodium (**A**), chloride (**B**), potassium (**C**), and calcium (**D**) in *S. canicula* after air exposure and recovery. Data are expressed as mean ± s.e.m. Asterisks (*) indicate significant differences between both groups at each time (*p* < 0.05, *n* = 7–8).

Air exposure also induced osmoregulatory disturbances in *S. canicula*, as reflected by impaired plasma levels of ions (Figure 2). Immediately after 18 min air exposure (time 0 h), plasma Cl^−^, K^+^ and Ca^2+^ concentrations increased when compared to undisturbed animals. The concentration of these ions returned to basal levels within the first 5 h of recovery. However, in the air-exposed group, plasma Na^+^ concentration only increased significantly at 5 h after the challenge. By using data from the present study (plasma Na^+^) and previously published in a parallel study (plasma urea) [32], but measured in the same samples, a significant negative correlation was determined between plasma Na^+^ and urea concentrations (r^2^ = 0.3412; *p* = 0.0004).

**Figure 3:**
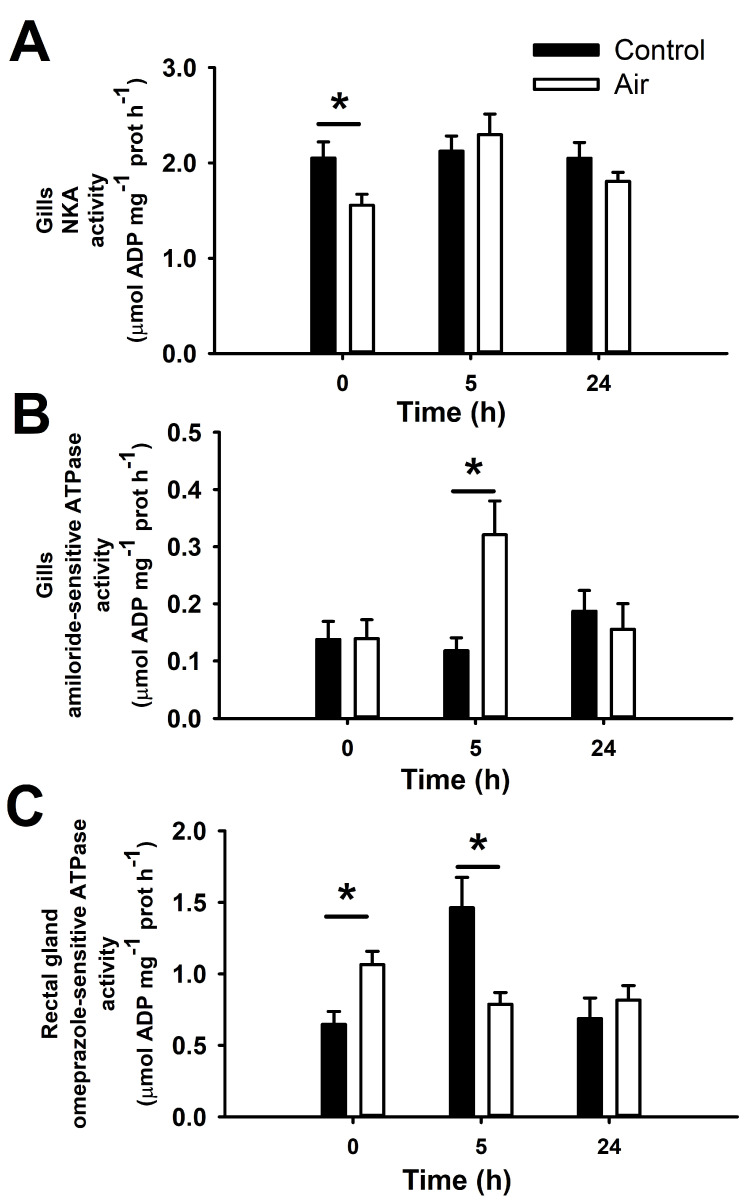
Gill Na^+^/K^+^-ATPase (NKA); (**A**), amiloride-sensitive ATPase (possibly a Na^+^/H^+^-exchanger from the NHE family); (**B**), and rectal gland omeprazole-sensitive ATPase (possibly a H^+^/K^+^-ATPase from the family HKA); and (**C**), activities in *S. canicula* after air exposure and recovery. Data are expressed as mean ± s.e.m. Asterisks (*) indicate significant differences between both groups at each time (*p* < 0.05, *n* = 7–8).

Some of these osmoregulatory disturbances matched impaired branchial NKA activity at time 0 h, which was significantly reduced in comparison to the control group (Figure 3). However, branchial VHA activity was not affected by air exposure, averaging 0.21 ± 0.02 and 0.20 ± 0.02 µmol ADP mg^−1^ prot h^−1^ in the control and air-exposed groups, respectively. In the gills, the activity of omeprazole-sensitive ATPases was below the level of detection, so we could not measure them in this tissue. The gills of air exposed fish 5 h after the challenge show an increased activity of some ATP -consuming enzyme(s) which were insensitive to ouabain, bafilomycin A1 and omeprazole (data not shown). 

Rectal gland omeprazole-sensitive ATPase (possibly an HKA enzyme and, therefore, it will be written as HKA from here on out) activity increased after 18 min of air-exposure (time 0 h), but decreased significantly 5 h later (Figure 3). In the control group, this activity increased at time 5 h, which may be associated to natural circadian rhythms. There were no significant changes in other ATPases activity measured in rectal gland or spiral valve. Thus, HKA activity in the spiral valve averaged 0.72 ± 0.09 and 0.73 ± 0.08 µmol ADP mg^−1^ prot h^−1^ in the control and air-exposed groups, respectively. In the rectal gland, NKA activity averaged 37.1 ± 1.4 and 36.9 ± 1.4 µmol ADP mg^−1^ prot h^−1^ in the control and air-exposed groups, respectively; and VHA activity averaged 0.24 ± 0.02 and 0.27 ± 0.04 µmol ADP mg^−1^ prot h^−1^ in the control and air-exposed groups, respectively. In the spiral valve, NKA activity averaged 1.82 ± 0.12 and 1.98 ± 0.15 µmol ADP mg^−1^ prot h^−1^ in the control and air-exposed groups, respectively; and VHA activity averaged 0.20 ± 0.02 and 0.23 ± 0.03 µmol ADP mg^−1^ prot h^−1^ in the control and air-exposed groups, respectively.

### 3.3. Activity of an Amiloride-Sensitive ATPase in Gills of S. canicula after Air Exposure

**Figure 4:**
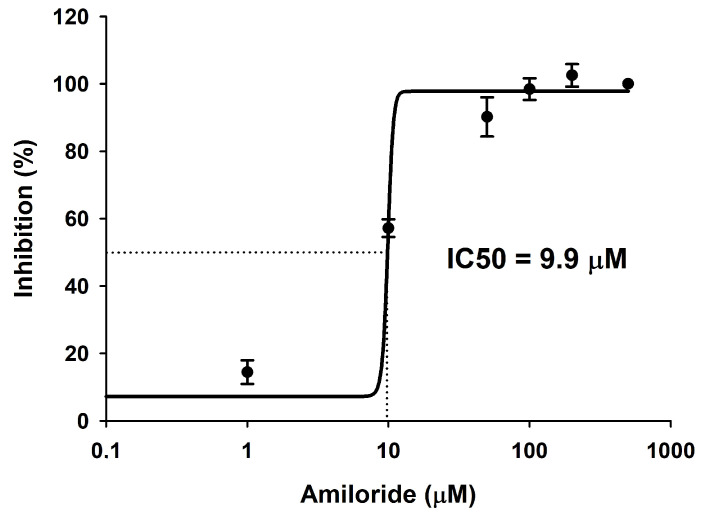
Biochemical inhibition of an ouabain/bafilomycin A1-insensitive ATPase enzyme in gills of *S. canicula* by amiloride. Dose-dependent inhibition of this ATPase activity by amiloride, and half-maximal inhibitory concentration (IC50), calculated with the best-fit equation was 9.9 µmol L^−1^. The data-points show mean ± s.e.m. from five independent assays in each of which amiloride-sensitive ATPase activity was measured in triplicate.

Based on observations of increased plasma Na^+^ and a slight decrease in plasma NH_4_^+^ in the air-exposed group, we hypothesized that the unknown ATP consuming mechanism may be associated with a member of the NHE family (NHE1, 2 or 3). To examine this, we conducted an in vitro assay with amiloride as an inhibitor of NHE enzymes [70]. Amiloride also inhibits Na^+^-channels, but they are not ATP-consuming molecules. However, as it was described that some NHEs are ATP-sensitive [69,71], NHE1 shows unique features compared to other NHEs. Thus, NHE1 was described as a phosphoprotein [68], and the only member of the NHE family that requires ATP to function while is also stimulated by cAMP [70,71]. With all this information, our biochemical approach is not capable of differentiating which enzyme(s) is being inhibited by amiloride, so our results focus on the description of an enzymatic mechanism that consumes ATP and is amiloride-sensitive and ouabain/bafilomycin A1-insensitive. Thus, by in vitro inhibiting gill homogenates with amiloride, we observed an ATP-consuming (ATPase) enzyme sensitive to this drug, postulated cautiously and provisionally until future studies demonstrate this, as an NHE in this species. Before routine measurements, a concentration-response curve was constructed in the range 1 µM to 500 µM. The concentration-response curve conforms to a typical sigmoidal inhibition curve with a calculated IC_50_ (the concentration in which 50% of the total enzymatic activity is inhibited) of 9.9 µM, and maximal effects at concentrations above 100 µM (Figure 4). 

Three NHE isoforms were described in branchial tissue of sharks and other elasmobranchs including NHE1 (present study), NHE2 and NHE3 [39,40,41,42]. Although NHE 1 and 3 isoforms are both sensitive to amiloride and ATP [71], NHE1 transport requires ATP and may be inhibited in the absence of cellular ATP [70], although its function as an ATPase was not previously described. Moreover, susceptibility to amiloride is isoform and species dependent [78,79]. With all this information, and since we are unable to recognize which enzyme we are describing in the present study, we will refer to these ATP-dependent amiloride-sensitive enzyme(s) as NHE. The amiloride-sensitive NHE activity was consistently measurable in gills of catshark at all sampling points, and enhanced in the air-exposed group at time 5 h (Figure 3). A significant positive correlation was determined between branchial NHE activity and plasma Na^+^ levels (r^2^ = 0.2432; *p* < 0.005), as well as between branchial NHE activity and the logarithm of plasma 1αOH-B (r^2^ = 0.1948; *p* < 0.005), where plasma 1αOH-B results are published in a parallel study [7] carried out with the same samples as the present one.

NHE activity was also observed in the rectal gland and in the spiral valve, though no changes occurred due to air exposure or during recovery time. The activity in the rectal gland averaged 0.60 ± 0.06 and 0.58 ± 0.04 µmol ADP mg^−1^ prot h^−1^ in the control and air-exposed groups, respectively. In the spiral valve, NHE activity averaged 0.44 ± 0.04 and 0.45 ± 0.04 µmol ADP mg^−1^ prot h^−1^ in the control and air-exposed groups, respectively.

### 3.4. Presence of nhe1 Transcripts in Gills of S. canicula

**Figure 5:**
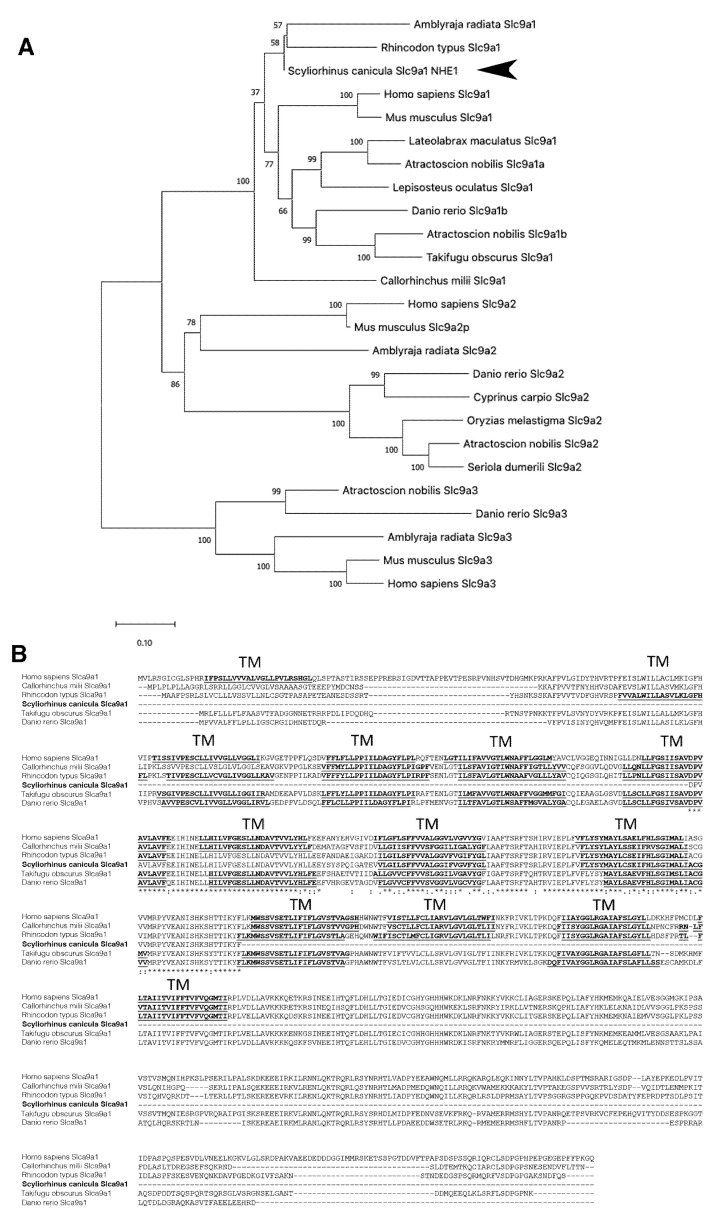
Phylogenetic and tree sequence analyses of slc9a1 (NHE1) from *S. canicula*. (**A**) Phylogenetic tree of the partial protein sequences of the identified small-spotted catshark NHE1 (indicated by black arrow) and other members of NHE family identified in other vertebrates. The phylogenetic tree was inferred using maximum likelihood method. The percentage of replicates trees in which the associated taxa clustered together in the bootstrap test (1000 replicates) are shown next to the branches. Scale bar refers to a phylogenetic distance of 0.10 amino acid substitutions per site. Accession numbers are: human *Homo sapiens*, NM_003047.5 slc9a1, XM_047445572.1, slc9a2, NM_001284351.3 slc9a3; house mouse *Mus musculus*, NP_058677.1 slc9a1, NP_001028461.2 slc9a2, NP_001074529.1 slc9a3; starry ray *Amblyraja radiata*, XP_032900998.1 slc9a1, XP_032878489.1 slc9a2, XP_032872420.1 slc9a3; whale shark *Rhincodon typus*, XM_020514167.1 slc9a1; Australian ghostshark *Callorhinchus milli*, XM_007894944.1 scl9a1, spotted gar *Lepisosteus oculatus*, XM_015349113.1 slc9a1; white weakfish *Atractoscion nobilis* MW962258.1 slc9a1a, MW962257.1 slc9a1b, MW962261.1 slc9a2, MW962259.1 slc9a3; obscure pufferfish *Takifugu obscurus*, AB200332.1 slc9a1; Japanese seabass *Lateolabrax maculatus*, MF481092.1 slc9a1; zebrafish *Danio rerio* NM_001113480.1 slc9a1b, EF591983.1 slc9a2, EF591984.1 slc9a3; Japanse rice fish *Oryzias melastigma*, XM_024286873.2 slc9a2; greater amberjack *Seriola dumerili*, XP_022596365.1 slc9a2; and common carp *Cyprinus carpio*, XM_042730904.1 slc9a2; (**B**) Multiple amino acid sequence alignments of slc9a1 from different vertebrate species. The transmembrane regions are shown in bold and underlined.

To further investigate the presence of NHE1 in the gills of catsharks, and since NHE2 and NHE3 have already been described in shark gills [78], we partially cloned the *nhe1* transcript in this tissue. The partial NHE1 fragment shared high homology with NHE1 of shark and ray species with identities between 95–91% and 91–80% when compared the proteins with other vertebrate species. These results were confirmed by the phylogenetic analysis that positioned *S. canicula* NHE1 protein within the corresponding NHE1 branch, completely separated from those branches representing NHE2 and NHE3 isoforms, showing more divergence with teleost sequences (Figure 5A). The cloning strategy developed in this study allowed us to obtain a partial *nhe1* fragment of 437 base pairs (bp), from which a 145 amino acid (aa) partial sequence was deduced. Two transmembrane domains relatively conserved were identified in the lesser spotted catshark NHE1 predicted protein (Figure 5B). 

### 3.5. Intermediary Metabolism in Osmoregulatory Tissues after Air Exposure

Activities of amino acid turnover-related enzymes (GDH, ALT and AST) in the gills and rectal gland are shown in Table 2. Gills showed higher activities of GDH and lower activities of ALT after 18 min of air exposure (time 0 h) compared to the control group, while an increase in AST activity occurred at time 5 h in the stressed group. In the rectal gland, GDH and ALT activities were lower at time 0 h in the air-exposed group, without further differences along the experimental time. However, AST activity in this tissue decreased in the air-exposed group at time 5 h after the stress, without any more differences between groups at times 0 h and 24 h.

The activity of glycolysis-related enzymes (HK, PK and G6PDH) in gill and rectal gland are shown in Table 2. Glycolysis pathway (lowered HK activity) seemed to be drastically decreased in the gills just after the air-exposure challenge. However, the activity of the HK enzyme in the gills was too low to be conclusive. No changes in the PK activity were described in gills at any time. The enzyme G6PDH revealed lower activity of the pentose shunt 5 h after the acute stress situation in catsharks, returning to basal levels at the end of the experimental period (24 h). Rectal gland HK and G6PDH activities did not show variations during the experiment in any group. However, PK activity decreased 5 h after the air-exposure compared to the control-undisturbed group.

All metabolites, ions and enzyme activities returned to basal levels at the end of the experiment (24 h).

### 3.6. In Vivo Effects of Amiloride on Plasma Parameters during Recovery after Air Exposure

**Figure 6:**
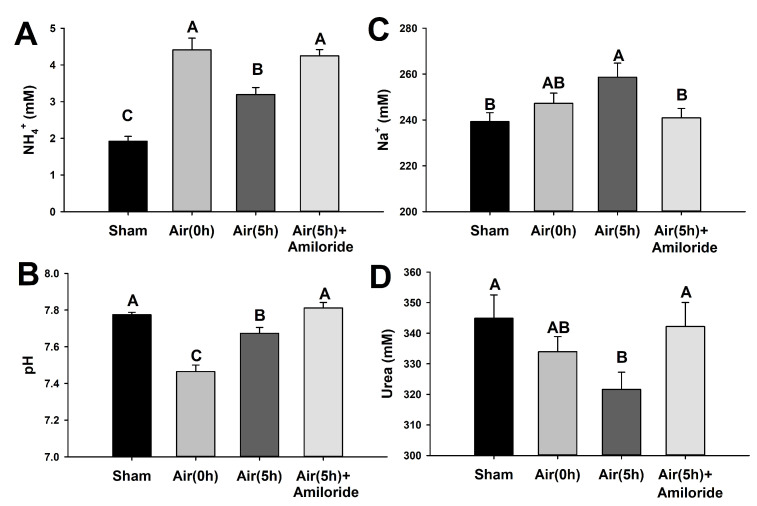
Plasma NH_4_^+^ (**A**), pH (**B**), Na^+^ (**C**), and urea (**D**) in *S. canicula* exposed to air and in vivo recovered in 100 µM amiloride for 5 h. The experiment includes an undisturbed-control group named “Sham”, a group sampled immediately after 18 min air exposure “Air(0h)”, and 5 h after recovery in control water “Air(5h)” or containing 100 µM amiloride “Air(5h)+Amiloride”. Data are expressed as mean ± s.e.m. Different letters indicate significantly different groups (*p* < 0.05, *n* = 12–14).

Amiloride effectively managed to maintain post-stress plasma NH_4_^+^ concentrations, with significant differences compared to the control group after 5 h recovery, while the latter showed significantly higher plasma NH_4_^+^ concentrations than the control-undisturbed group (Figure 6). Similar to the previous air-exposure and recovery experiment (Figure 2), plasma Na^+^ concentration increased after 5 h of recovery, a trend seen to be more pronounced in the absence of amiloride in the water (Figure 6). Amiloride treatment led to a return in plasma Na^+^ to control-undisturbed levels after 5 h recovery. Plasma urea concentration, similar to a paralleled study [32], significantly decreased after 5 h recovery, but returned to basal levels in the presence of amiloride. In our study, a significant negative correlation was determined between plasma Na^+^ and urea concentrations in those animals not treated with amiloride (r^2^ = 0.3707; *p* = 0.0012), while those treated with amiloride and after 5 h recovery show a positive correlation between these variables (r^2^ = 0.2282; *p* = 0.0841). Plasma pH drastically decreased immediately after 18 min air exposure (from pH 7.77 to 7.46), and significantly increased after 5 h recovery in previous control conditions (reaching pH 7.67) though with differences with the control-undisturbed group. In the presence of amiloride, plasma pH returned to basal levels after 5 h recovery (Figure 6).

Plasma HCO_3_^−^, Cl^−^, Ca^2+^, osmolality, and phosphate after air-exposure and further in vivo recovery with or without amiloride are shown in Table 3. HCO_3_^−^ increased immediately after air exposure and maintain that concentration 5 h later in the absence of amiloride, while significantly increased in its presence. Plasma chloride increased immediately after air exposure, as described in the previous experiment, and returned to control values with or without amiloride. Plasma calcium levels only showed changes when *S. canicula* is recovered during 5 h in the presence of amiloride, reducing its concentration significantly. Plasma osmolality, significantly increased immediately after air exposure but returned to control values after 5 h recovery (with and without amiloride). No changes in plasma phosphate are described in this study.

Plasma energy metabolites of the in vivo response to amiloride after air exposure experiment are shown in Table 3. No changes are described in free proteins. However, plasma free amino acids significantly decreased after 5 h recovery in the absence of amiloride, while amiloride managed to almost return them to the control-undisturbed group values. Plasma glucose increased after 5 h recovery in the absence of amiloride, but slightly returned to control values in the presence of this drug. Plasma lactate concentration increased in all experimental groups compared to control-undisturbed fish.

### 3.7. Ex Vivo Incubation of Branchial Explants and Amiloride-Sensitive ATPase Activity

**Figure 7:**
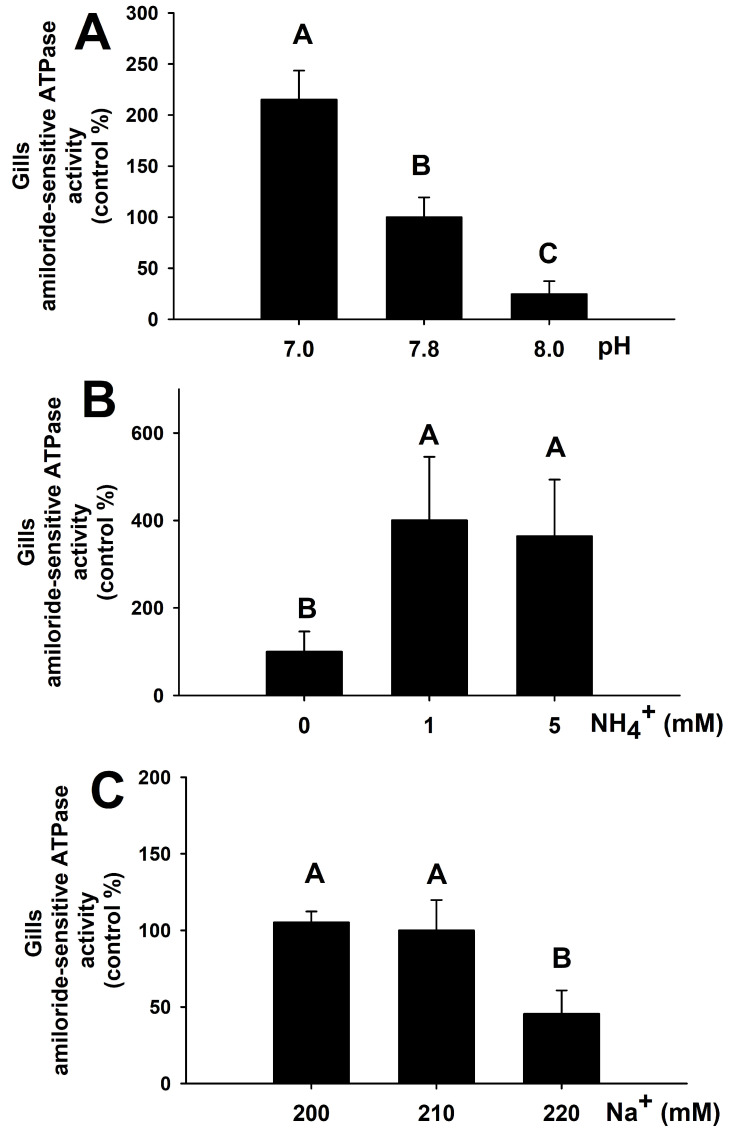
Amiloride-sensitive ATPase activity, putatively associated to a Na^+^/H^+^(NH_4_^+^)-exchanger (NHE) (expressed as control %), in ex vivo gill explants *of S. canicula* incubated at different pH (**A**), or NH_4_^+^ (**B**) and Na^+^ (**C**) concentrations. Data are expressed as mean ± s.e.m. Different letters indicate significantly different groups (*p* < 0.05, *n* = 8).

After ex vivo incubation of *S. canicula* gill explants, the effect of pH, NH_4_^+^, and Na^+^ on amiloride-sensitive ATPase (associated to a NHE enzyme) activity is shown in Figure 7. Averaged activity for all control groups was 0.18 ± 0.03 µmol ADP mg prot^−1^ h^−1^. Increased pH diminished the suggested gill amiloride-sensitive ATPase activity significantly, reaching the lowest rates at pH 8.0 and the highest at pH 7.0, with a 2-fold increase in the latter condition compared to pH 7.8. The presence of NH_4_^+^ in the incubation medium above 1 mM induced a 4-fold increase in amiloride-sensitive ATPase activity compared to those gill explants incubated in the absence of ammonium. When gill explants are ex vivo incubated for 5 h at 220 mM Na^+^, which is the plasma concentration of *S. canicula* after 5 h recovery following an air exposure challenge, the putative NHE activity decreased to half its basal values. Branchial NKA activity in all ex vivo experiments show no changes due to pH, NH_4_^+^, or Na^+^, and ranged from 1.96 to 3.72 µmol ADP mg prot^−1^ h^−1^ (data not shown).

## 4. Discussion

In this study, we describe the main processes of amino acid catabolism after an episode of acute stress (air exposure) in an elasmobranch, the lesser spotted catshark (*S. canicula*). White muscle appears to be the main tissue for energy consumption, while liver also oxidizes amino acids, producing NH_4_^+^. Air exposure in this species also generates osmoregulatory imbalances, requiring the gills and rectal gland to play an important role in restoring homeostasis. 

### 4.1. Acute-Stress Induces Amino Acids Catabolism in Catsharks

As expected, *S. canicula* appears to utilize free amino acids to fuel the energy demand imposed by an acute-stress challenge, as seen by their lower concentrations in white muscle and plasma after air exposure. The evolutionary strategy of oxidizing amino acids after short-term stressors was observed in the leopard shark (*Triakis semifasciata*), which modified its intermediary metabolism towards amino acid catabolism after 48 h at different environmental salinities [80], and in the epaulette shark (*Hemiscyllium ocellatum*) facing hypoxia for less than 48 h [81]. However, other information is scarce as few studies have examined amino acid catabolism in shorter periods in elasmobranchs. The relevance of amino acid turnover in white muscle of elasmobranchs has been wonderfully explained in several review articles [18,19,20]. These papers describe the importance of the liver as an important site of amino acid oxidation, as it is in teleosts [19,82], holostei [83], and dipnoi [84]. Ammonium is produced after amino acid catabolism in sharks, altogether with other nitrogenous substrates [19]. In this sense, *S. acanthias* under food deprivation enhanced plasma NH_4_^+^ levels after its release from white muscle resembling amino acid oxidation due to starving [19], while *S. canicula* greatly increased plasma NH_4_^+^ after air exposure (present study).

In elasmobranchs, GDH [27], AST [19], and other transaminases are associated with enhanced amino acid turnover. In our study, amino acids seemed to play an important role in the physiological recovery from air exposure, as indicated by the observed changes in GDH, AST, and ALT activities in white muscle and liver. These are the main tissues managing energy metabolism in sharks, but osmoregulatory tissues (gills and rectal gland) should also be considered, as in situ amino acid oxidation may be employed by them to partially fuel ionoregulatory processes [82,84]. Moreover, rate limiting enzymes of glycolysis and the pentose phosphate shunt were also modified in these tissues (present study), suggesting the importance of carbohydrates as energy metabolites during an air-exposure challenge in *S. canicula* [7].

### 4.2. Osmoregulatory Imbalances after an Air-Exposure Challenge

Air exposure in *S. canicula* is immediately followed by an increase in plasma Cl^−^, K^+^, and Ca^2+^ concentrations (but not in Na^+^ levels), concomitantly with higher plasma osmolality (present study) and lower muscle water content [43]. Concentration of ions in the blood after an acute challenge is a paradigmatic secondary stress response in marine elasmobranchs [10,15,16,85] and teleosts [86] due to increased permeability of the branchial epithelium, and imbalances of active ion-transporters in osmoregulatory tissues. Our results in *S. canicula* showed that branchial NKA activity decreased after air exposure in agreement with what was described in teleosts [35]. In the present study, gill NKA activity as well as plasma osmolality, and Cl^−^, K^+^, and Ca^2+^ concentrations recovered their homeostatic levels in less than 5 h. However, Na^+^ balance followed a completely different pattern, with a delayed increase and maximum plasma levels 5 h after air exposure.

With data from a parallel study obtained using the same samples as the present study [7], we highlight a positive correlation between plasma Na^+^ and 1αOH-B values in *S. canicula* following air exposure. In this species, 1αOH-B maintains plasma Na^+^ concentrations, minimizing its excretion via osmoregulatory tissues (gills and rectal gland) [12]. Moreover, the present study confirms that in *S. canicula* plasma Na^+^ and urea levels are negatively correlated, as described before in specimens of the same species maintained in seawater [13]. In sharks, the presence of a branchial ATP-dependent Na^+^/urea-antiporter has been proposed [20] which could be related to the balance between both osmolytes. In *S. acanthias*, gills account for 91.2% of nitrogen excretion (mostly urea, but also NH_4_^+^) [22], increasing the urea efflux after acute-stress [17]. Thus, a drastic decrease in plasma urea occurs after 5 h recovery following air exposure in *S. canicula*. Urea excretion is also stimulated by the presence of NH_4_^+^ in the blood (produced during amino acid catabolism), as demonstrated in in vivo experiments with cannulated *S. acanthias* [22]. Our data indicated that *S. canicula* maintains, after air exposure, circulating osmotic pressure through the synchronous regulation of urea and Na^+^.

In this sense, the present study postulated two novel ATPase enzymes in osmoregulatory tissues of sharks: (i) an amiloride-sensitive ATPase (which may be cautiously associated with an NHE enzyme), and (ii) an omeprazole-sensitive ATPase (which could be a member of the HKA family). We observed the highest branchial NHE activity and plasma Na^+^ levels 5 h after exposure to air, matching lowered plasma NH_4_^+^. Our hypothesis is that branchial amiloride-sensitive ATPase participates in NH_4_^+^ excretion through Na^+^ exchange, although more studies are required to confirm it.

The presence of a Na^+^/H^+^-exchanger type 1 (NHE1) in gills of *S. canicula* has been proposed based on molecular evidence (a partial transcript of the *nhe1* is described here, matching its sequence to other *nhe1* sequences from other vertebrates, and revealed as different from *nhe2* and *nhe3* sequences). In vitro incubation of gills detected an ATP-consuming ouabain/bafilomycin A1-insensitive and amiloride-sensitive enzyme with biochemical resemblances to the mammalian NHE1, although our biochemical approach cannot differentiate between amiloride-sensitive enzymes. Amiloride inhibits NHEs and Na^+^-channels [62], and those NHE isoforms described in gills of sharks (NHE 1 to 3) are sensitive to ATP [71,87]. In the gills of catsharks, the protein we cautiously postulate here as a NHE presents a calculated IC_50_ for amiloride of 9.9 µM, which is similar to the described IC_50_ in human and rat NHE1 (1–3 µM), while falling within the range of other NHEs (1 to 100 µM) from different mammalian species [88,89]. We hypothesized that branquial NHE has a role in NH_4_^+^ excretion in sharks, as occurs in mammalian NHE1 [70], as well as NHE2 and NHE3 in some teleosts [90]. To investigate these roles, we conducted a series of in vivo and ex vivo experiments (described below).

The rectal gland regulates circulating Na^+^, Cl^−^, and, to a lower extent K^+^, and its activity depends on blood pH [29]. Therefore, the drastic blood acidification after air exposure observed in our study (Figure 6) might explain the changes in omeprazole-sensitive ATPase (cautiously addressed here as HKA) activity. As the rectal gland has a negligible role in systemic acid-base balance due to the low HCO_3_^−^ content of its secreted fluid [29], our results suggest that HKA could be involved in the regulation of plasma K^+^. However, further studies are encouraged to explore the blood buffering capacity of this enzyme as it excretes H^+^. Omeprazole-sensitive HKA was classically related to gastric acidification [91], but the present study is the second to describe this enzyme in non-gastric tissues of vertebrates, along with the rectum of the teleost Senegalese sole (*Solea senegalensis*), where it’s role is related to osmoregulation [61]. Other authors described a putative HKA in gills of the Atlantic stingray (*Dasyatis sabina*) that may be associated with freshwater acclimation [38], albeit the lack of HKA activity in gills of *S. canicula* observed in our study may indicate, as an hypothesis, that the transcript from *D. sabina* did not correspond to an omeprazole-sensitive ATPase. In our study, the spiral valve seems not to act as an important osmoregulatory tissue after acute stress, as previously described in other sharks [27]. Furthermore, the high NKA activity in rectal gland (having rates 20-times higher than observed in the gills) did not change due to air exposure in *S. canicula*.

### 4.3. In Vivo Amiloride Inhibition after Air Exposure and Recovery

We confirm that amiloride-sensitive elements (including branchial NHEs) are involved in the regulation of blood NH_4_^+^, Na^+^, urea, and pH after an acute-stress challenge. Amiloride strongly inhibits NHEs and Na^+^-channels (showing IC_50_ from 0.34 to 1 µM), but it also inhibits Na^+^/Ca^2+^-exchangers, albeit weakly, with IC_50_ above 1.1 mM [62]. Thus, recovery of *S. canicula* exposed to air in seawater with or without 100 µM amiloride, evaluates the effect of inhibiting branchial Na^+^-channels and NHEs. As postulated, inhibiting NHEs failed to decrease the high plasma NH_4_^+^ concentrations after air exposure. With our in vivo experimental approach, we were unable to determine if NHE1, localized to the basolateral membrane of polarized epithelial cells [70], was completely inhibited in gills of *S. canicula*. However, as there are other NHE isoforms (NHE2 and NHE3) described in branchial epithelial cells of several shark species [42], the present study cannot differentiate which NHE enzymes have been inhibited and to what extent. In any case, and to the best of our knowledge, our study suggests (for the first time) branchial NHEs as a novel NH_4_^+^ excretion mechanism in elasmobranchs.

In *S. canicula*, air exposure induced a ~20 mM plasma Na^+^ increase after 5 h (compensated through a stoichiometric ~20 mM plasma urea decrease), but amiloride successfully managed to inhibit Na^+^ uptake, maintaining basal plasma Na^+^ (and urea) concentrations. This supports the idea that plasma Na^+^ and urea are mutually and inversely regulated to maintain osmotic balance. However, further studies are required to better describe the processes controlling levels of both osmolytes in elasmobranchs, including branchial transporters such as the Na^+^/urea exchanger [20,33].

Our work also shows a drastic drop in plasma pH due to air exposure, similar to that described in nursehound (*Scyliorhinus stellaris*) and Atlantic sharpnose (*Rhizoprionodon terraenovae*) sharks after exhausting activity [31,92]. In fish, acid-base balance is mostly regulated by their gills [93]. While amiloride inhibits branchial NHEs, which usually secrete H^+^ in exchange of Na^+^ [70], stressed *S. canicula* in the presence of amiloride managed to recover plasma pH values, suggesting the action of alternative mechanisms for acid-base regulation in this species. In this regard, the 2-fold rise in plasma HCO_3_^−^ concentration due to amiloride is an expected buffering response [93]. In teleosts, blood acidosis is compensated with increased plasma HCO_3_^−^ after air exposure [35], and during a drop in environmental pH [94,95]. In teleosts, carbonic anhydrases catalyzed the formation of intracellular HCO_3_^−^, regulating acid-base balance [93], but the mechanisms underlying this regulation in elasmobranchs are still not well understood.

### 4.4. Ex Vivo Characterization of an Amiloride-Sensitive ATPase (NHE) Activity

By using an ex vivo culture of branchial explants, this study suggests that amiloride-sensitive ATPase (hypothetised as a NHE enzyme) activity may be regulated by pH, and Na^+^ and NH_4_^+^ concentrations. Low pH mimicking plasma acidosis after air exposure stimulated gill NHE activity, as expected in an enzyme excreting H^+^ to regulate acid-base balance. Low Na^+^ concentrations upregulated NHE activity, increasing Na^+^ uptake. Both H^+^ excretion and Na^+^ uptake are the known functions of this enzyme in vertebrates [70]. NH_4_^+^ managed to increase branchial NHE activity in *S. canicula* at concentrations above 1 mM. Thus, an alternative pathway of NH_4_^+^ excretion through NHEs located in the branchial epithelium of elasmobranchs is described herein, reinforcing the importance of the gills as the predominant site for this action [22,28].

### 4.5. Sharks Emerge as Unexpected Study Models Due to Their Similarities with Mammals 

This is the first time that an amiloride-sensitive putative Na^+^/H^+^-exchanger (NHE), is described in gills of any elasmobranch. Here, we describe for the first time a NHE1 isoform in gills of an elasmobranch, although this transporter appears in different tissues of marine [96] and freshwater teleosts [97], including the gills [66,67]. In some teleosts, NHE2 and NHE3 (but not NHE1) are involved in ammonia excretion through the gills [90] or the intestine [98] by substituting NH_4_^+^ for H^+^ [67]. Unless NHE2- and NHE3-like exchangers are present in gills of elasmobranchs [42], their role in ammonia transport was not suggested before. Thus, our study suggests NHEs as potential NH_4_^+^ transporters in elasmobranchs, resembling that of mice and humans [70]. However, this hypothesis should be further confirmed.

There are great similarities in the homology of NHEs between elasmobranchs and mammals [99] as observed for the NHE2 and 3 in Figure 5. Therefore, branchial explants of these species may be employed as a useful ex vivo model to study acid-base and Na^+^ regulation in mammals. In this sense, the striking resemblance of teleost gills with the mammalian kidney has arisen as a powerful tool to study ion transport in humans [100]. In the same way, we postulate that sharks could be a useful alternative model to study NHEs in comparison to other vertebrates. Interestingly, certain carcinogenic processes are related to intracellular pH imbalances, and NHE1 has been identified as an important element that can trigger tumor responses [101]. We also believe it would be interesting to continue the research on NH_4_^+^/Na^+^ exchange in sharks as a relevant process in ureotelic animals.

## 5. Conclusions

Acute stress situations, such as air exposure, in *S. canicula* increase protein catabolism to meet the challenge. Under this situation, amino acids are consumed in white muscle and liver, generating ammonia. Moreover, a series of osmotic imbalances also occur that require a coordinated work of both gills and rectal gland to restore homeostasis. Future studies should focus on describing, in detail, the mechanisms related to ammonium excretion and acid-base balance recovery in elasmobranchs.

## Figures and Tables

**Table 1:** Activity of enzymes involved in the intermediary metabolism after air exposure in white muscle and liver *of S. canicula*. Changes in amino acid turnover-related enzymes activities (glutamate dehydrogenase, GDH; aspartate aminotransferase, AST; and alanine aminotransferase, ALT) activities (in U mg^−1^ prot) in white muscle and liver of *S. canicula* after air exposure. Data are expressed as mean ± s.e.m. Asterisks (*) indicate significant differences between both groups at each sampling time (*p* < 0.05, *n* = 7–8).

Tissue	Enzyme act. (U mg^−1^ Prot)	Group	0 h	5 h	24 h
Muscle	GDH	Control	1.25 ± 0.49	2.27 ± 0.51	2.86 ± 0.30
		Air	0.94 ± 0.44	0.39 ± 0.24 *	2.28 ± 0.42
	AST	Control	0.84 ± 0.17	0.69 ± 0.12	1.33 ± 0.12
		Air	0.31 ± 0.06 *	0.57 ± 0.18	1.13 ± 0.18
	ALT	Control	0.21 ± 0.04	0.31 ± 0.06	0.23 ± 0.06
		Air	0.16 ± 0.04	0.30 ± 0.08	0.25 ± 0.06
Liver	GDH	Control	6.94 ± 0.53	8.38 ± 0.43	7.84 ± 0.38
		Air	8.11 ± 0.36	6.51 ± 0.45 *	8.65 ± 0.26
	AST	Control	1.22 ± 0.12	0.78 ± 0.19	1.07 ± 0.12
		Air	1.27 ± 0.14	0.88 ± 0.15	1.44 ± 0.14
	ALT	Control	3.39 ± 0.27	3.60 ± 0.26	3.97 ± 0.29
		Air	3.42 ± 0.18	3.09 ± 0.22	3.69 ± 0.21

**Table 2:** Activity of enzymes involved in the intermediary metabolism after air exposure in gills and rectal gland *of S. canicula*. Changes in amino acid turnover-related and glycolytic-related enzymes (hexokinase, HK; pyruvate kinase, PK; and glucose-6-phosphate dehydrogenase, G6PDH) activities (in U mg^−1^ prot) in gills and rectal gland of *S. canicula* after air exposure. Data are expressed as mean ± s.e.m. Asterisks (*) indicate significant differences between both groups at each sampling time (*p* < 0.05, *n* = 7–8).

Tissue	Enzyme act. (U mg^−1^ Prot)	Group	0 h	5 h	24 h
Gills	GDH	Control	0.01 ± 0.00	0.01 ± 0.00	0.02 ± 0.00
		Air	0.02 ± 0.01 *	0.01 ± 0.00	0.02 ± 0.00
	AST	Control	0.26 ± 0.12	0.54 ± 0.17	0.25 ± 0.07
		Air	0.31 ± 0.09	1.07 ± 0.26 *	0.60 ± 0.18
	ALT	Control	0.50 ± 0.08	0.31 ± 0.05	0.54 ± 0.05
		Air	0.22 ± 0.06 *	0.46 ± 0.05	0.46 ± 0.07
	HK	Control	0.02 ± 0.00	0.02 ± 0.00	0.02 ± 0.00
		Air	0.00 ± 0.00 *	0.02 ± 0.00	0.01 ± 0.00
	PK	Control	0.04 ± 0.01	0.06 ± 0.01	0.07 ± 0.02
		Air	0.06 ± 0.00	0.07 ± 0.01	0.06 ± 0.00
	G6PDH	Control	0.01 ± 0.00	0.02 ± 0.00	0.01 ± 0.00
		Air	0.01 ± 0.00	0.01 ± 0.00 *	0.01 ± 0.00
Rectal gland	GDH	Control	5.13 ± 0.54	4.49 ± 0.35	5.06 ± 0.46
		Air	3.98 ± 0.25 *	4.04 ± 0.16	4.00 ± 0.39
	AST	Control	32.7 ± 3.4	40.3 ± 1.8	34.4 ± 4.4
		Air	37.0 ± 1.4	30.2 ± 2.2*	32.4 ± 3.3
	ALT	Control	0.66 ± 0.10	0.43 ± 0.05	0.45 ± 0.08
		Air	0.34 ± 0.04 *	0.24 ± 0.08	0.28 ± 0.04
	HK	Control	1.81 ± 0.13	1.55 ± 0.11	1.57 ± 0.09
		Air	1.85 ± 0.16	1.35 ± 0.11	1.47 ± 0.18
	PK	Control	23.9 ± 1.0	22.1 ± 0.5	20.3 ± 1.8
		Air	23.5 ± 1.6	17.0 ± 0.8 *	17.1 ± 1.2
	G6PDH	Control	1.89 ± 0.21	1.27 ± 0.18	1.44 ± 0.16
		Air	1.46 ± 0.16	1.31 ± 0.20	1.20 ± 0.06

**Table 3:** Plasma parameters in *S. canicula* exposed to air and in vivo recovered in 100 µM amiloride for 5 h. The experiment includes undisturbed-control fish (Sham), a group sampled immediately after 18 min air exposure (Air 0 h), and 5 h after recovery in control water (Air 5 h) or containing 100 µM amiloride (Air 5 h + Amil.). Data are expressed as mean ± s.e.m. Different letters indicate significantly different groups (*p* < 0.05, one-way ANOVA followed by a Tukey’s post hoc test, *n* = 12–14).

Plasma Parameter	Sham	Air 0 h	Air 5 h	Air 5 h + Amil.
HCO_3_^−^ (mM)	3.39 ± 0.18 C	5.50 ± 0.40 B	5.58 ± 0.25 B	6.25 ± 0.27 A
Cl^−^ (mM)	295 ± 2 B	306 ± 4 A	295 ± 4 B	294 ± 3 B
Ca^2+^ (mM)	4.1 ± 0.1 A	4.1 ± 0.1 A	4.0 ± 0.1 AB	3.5 ± 0.1 B
Osmolality (mOsm kg^−1^)	957 ± 6 B	996 ± 8 A	986 ± 10 AB	982 ± 8 AB
Phosphate (mM)	1.80 ± 0.19	2.10 ± 0.24	2.40 ± 0.23	1.93 ± 0.18
Proteins (mg dL^−1^)	28.2 ± 0.8	28.6 ± 1.3	26.9 ± 0.9	30.0 ± 0.8
Amino acids (mM)	12.3 ± 0.9 A	10.6 ± 1.0 AB	7.8 ± 0.6 B	10.8 ± 0.9 AB
Glucose (mM)	4.3 ± 0.6 B	5.8 ± 0.4 AB	6.7 ± 0.5 A	5.3 ± 0.5 AB
Lactate (mM)	0.58 ± 0.07 B	7.33 ± 0.95 A	7.64 ± 0.94 A	4.94 ± 0.74 A

## Data Availability

The data presented in this study are available on request from the corresponding author.

## Supplementary Materials

The following supporting information can be downloaded at https://www.mdpi.com/article/10.3390/ani12091192/s1. Table S1: Sequence of degenerate primers used for *nhe1* cloning. Table S2: *p*-values from two-way ANOVA of parameters measured in plasma, liver, muscle, gills, rectal gland, and spiral valve of *S. canicula* in a time-course experiment following air exposure.
