# Supplementary material for: Acute Stress in Lesser-Spotted Catshark (Scyliorhinus canicula Linnaeus, 1758) Promotes Amino Acid Catabolism and Osmoregulatory Imbalances
Source: Animals (Basel). 2022 May 6;12(9):1192. doi: 10.3390/ani12091192 (PMC9105869; doi:10.3390/ani12091192)

Acute stress in lesser-spotted catshark (*Scyliorhinus canicula* Linnaeus, 1758) promotes amino acid catabolism and osmoregulatory imbalances

Ignacio Ruiz-Jarabo, José A. Paullada-Salmerón, Ismael Jerez-Cepa, José Belquior Gonçalves Neto, Jason S. Bystriansky and Juan M. Mancera

SUPPLEMENTARY MATERIAL

Table S1. Sequence of degenerate primers used for *nhe1* cloning.

Gene Molecular cloning	Primer name	5` to 3` sequence	Temperature
Nhe1 (Slc9a1)	***Fnhe1.1***	fw: CCATCATTCTGGATGCTGGCTAC	60.1ºC
	Rnhe1.1	rv: GTCCACGGCMGAGATGATG	56.9ºC
	Fnhe1.2	fw: CATCATCTCKGCCGTGGAC	56.9ºC
	Rnhe1.2	rv: CTCGCTSACRCTGCTCCACAT	60.9ºC
	Fnhe1.3	fw: ATGTGGAGCAGYGTSAGCGAG	60.9ºC
	Rnhe1.3	rv: CGRATGGTCATSCCCTGMAC	58.0ºC
	Fnhe1.4	fw: GTKCAGGGSATGACCATYCG	58.0ºC
	Rnhe1.4	rv: TGTGRATYCYTCATTGATGG	51.2ºC

Forward (fw) and reverse (rv) primers.

Table S2. P-values from two-way ANOVA of parameters measured in plasma, liver, muscle, gills, rectal gland and spiral valve of *S. canicula* in a time-course experiment following air exposure. Group (control and air-exposed) and time (0, 5 and 24 h) are the main factors. *NS* not significant (p > 0.1).

Tissue	Parameter	Group	Time	Group*Time
**Plasma**	Proteins	*NS*	*NS*	*NS*
	Amino acids	<0.005	<0.02	<0.02
	NH_4^+	<0.005	*NS*	*NS*
	Na^+	<0.03	<0.01	*NS*
	Cl^-	<0.005	*NS*	<0.005
	K^+	<0.05	<0.000001	<0.00005
	Ca^{2+}	0.074	<0.02	<0.03
**Liver**	Amino acids	*NS*	*NS*	*NS*
	GDH	*NS*	*NS*	<0.002
	AST	*NS*	<0.01	*NS*
	ALT	*NS*	*NS*	*NS*
**Muscle**	Amino acids	<0.005	<0.00001	*NS*
	GDH	<0.01	<0.002	*NS*
	AST	<0.03	<0.0001	*NS*
	ALT	*NS*	*NS*	*NS*
**Gills**	NKA	*NS*	<0.05	*NS*
	NHE	0.085	*NS*	<0.05
	HA	*NS*	*NS*	*NS*
	GDH	<0.05	<0.03	*NS*
	AST	<0.03	<0.01	*NS*
	ALT	*NS*	0.063	<0.01
	HK	<0.0005	<0.0001	<0.005
	PK	*NS*	*NS*	*NS*
	G6PDH	0.062	*NS*	<0.01
**Rectal gland**	NKA	*NS*	*NS*	*NS*
	NHE	*NS*	<0.001	*NS*
	HA	*NS*	<0.005	*NS*
	HKA	*NS*	<0.05	<0.0005
	GDH	<0.01	*NS*	*NS*
	AST	*NS*	*NS*	*NS*
	ALT	<0.0005	0.063	*NS*
	HK	*NS*	<0.05	*NS*
	PK	<0.01	<0.0005	*NS*
	G6PDH	*NS*	0.061	*NS*
**Spiral valve**	NKA	*NS*	*NS*	*NS*
	NHE	*NS*	0.054	*NS*
	HA	*NS*	*NS*	*NS*
	HKA	*NS*	*NS*	0.058

Figure S1. Measured and theoretical NH_4^+ concentrations in incubation medium (plasma-like medium) and milli Q water (MQw). Each dot represents 4 independent measurements for each concentration (mean ± SEM).

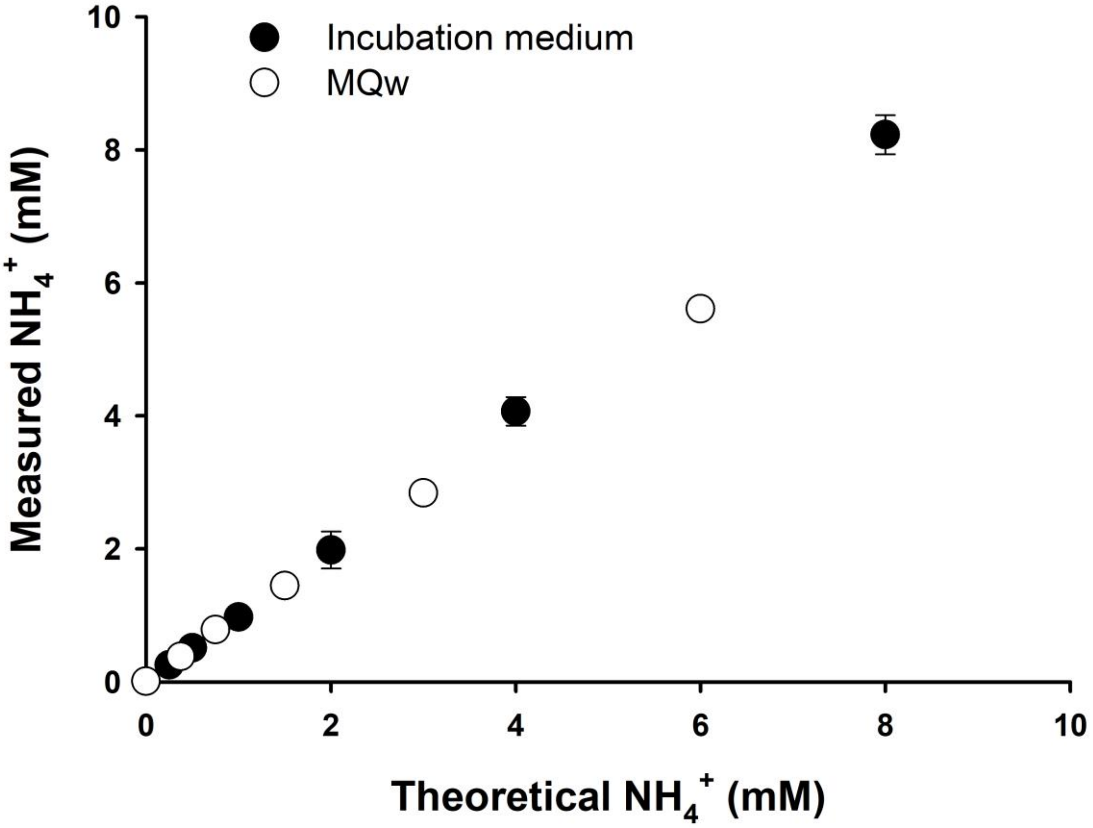

Supplement: Supplementary file 1 [file animals-12-01192-s001.zip › animals-1658366-supplementary.pdf]
